# Analytical Criticalities Associated to Different Immunological Methods for Serum Free Light Chain Detection in Plasma Cell Dyscrasias: A Description of Particular Clinical Cases

**DOI:** 10.3390/ijms18040804

**Published:** 2017-04-12

**Authors:** Rocco Sabatino, Antonio Perrone, Marco Cuomo, Sandra Liotti, Vittoria Barchiesi, Monica Cantile, Ernesta Cavalcanti

**Affiliations:** 1 Division of Laboratory Medicine, Department of Pathology and Laboratory Diagnostics, Istituto Nazionale Tumori “Fondazione G. Pascale”, IRCCS, Naples 80131, Italy; roc.sabatino@gmail.com (R.S.); perrone.antonio85@gmail.com (A.P.); m.cuomo@istitutotumori.na.it (M.C.); s.liotti@istitutotumori.na.it (S.L.); v.barchiesi@istitutotumori.na.it (V.B.); 2 Pathology Unit, Department of Pathology and Laboratory Diagnostics, Istituto Nazionale Tumori “Fondazione G. Pascale”, IRCCS, Naples 80131, Italy; m.cantile@istitutotumori.na.it

**Keywords:** plasma cell dyscrasias, multiple myeloma, serum free light chains

## Abstract

Current criteria for differential diagnosis of multiple myeloma (MM), Monoclonal gammopathy of undetermined significance (MGUS), and smoldering multiple myeloma (SMM) are included in the 2003 guidelines by the International Myeloma Working Group (IMWG). An updated version was then published in 2014, highlighting the importance of serum free light chain (sFLC) detection, as well as the κ/λ ratio as excellent indicators of clonality. At present, two commercial assays for sFLC quantification are available: the Freelite™ assay and the N-Latex assay. The first was developed by The Binding Site based on a mixture of polyclonal antibodies directed against a variety of FLC epitopes. It may be run on a wide range of nephelometers, as well as on turbidimeters. The second method was developed by Siemens and runs exclusively on Siemens instruments. It employs a probe mixture of mouse monoclonal antibodies. The aim of our study was to evaluate sFLC measurement and calculated κ/λ ratio in 85 patients with monoclonal gammopathies (MGs) in order to compare methods. We demonstrated that there is only a moderate concordance between the two FLC assays. In particular, in one case, we observed no qualitative alterations of the serum protein pattern, and in the absence of a Freelite™ assay, sFLC measurement would not have been possible to highlight the increase of λ FLC.

## 1. Introduction

Monoclonal gammopathy (MG) or plasma cells dyscrasias are disorders characterized by the proliferation and accumulation of plasma cell clone synthesizing immunoglobulin with identical isotopic and idiotipic features, detectable by serum or urine electrophoresis commonly referred to as a monoclonal protein (M-protein). The presence of an M-protein is associated with the majority of MG. Among these, multiple myeloma (MM) is a neoplastic disease characterized by the expansion of a B-cell clone, accounting for approximately 0.8% of all types of malignancies in the world and about 1% in Europe [1,2]. MM is generally preceded by monoclonal gammopathy of undetermined significance (MGUS) and smoldering multiple myeloma (SMM), which is considered a clinically-defined intermediate stage. [3,4]. MGUS accounts for over 50% of detected M-protein, whereas 35% of M-protein are due to multiple myeloma (MM), 10% to amyloidosis (AL), and the remaining 5% are associated with rare conditions such as cryoglobulinemia [5,6].

According to guidelines, one of the mainstays concerning the risk of progression is determined by the type of M-protein involved, its entity, as well as the ratio between κ and λ serum free light chain (sFLC), along with the presence of immunoparesis and medullary plasmacytosis [7,8,9,10,11,12].

The current criteria for differential diagnosis between MM, MGUS, and SMM were defined in 2003 [13] and revised with few modifications in 2009 [14]. Serum immunofixation (sIFE), serum protein electrophoresis (SPE), and sFLC combined with urine immunofixation (uIFE) tests are actually considered the golden standard for the screening of MG. Indeed, the combination of these methods provides the highest sensitivity (98.6%) for the detection of MGs [15,16]. 

In 2014, they have been updated again through the International Myeloma Working Group (IMWG) guidelines [17] that included sFLC evaluation and a calculated κ/λ ratio as biomarkers of malignancy. The evaluation of these parameters are recommended for patient management, including screening, prognosis, therapy, and patient monitoring, as well as for the diagnosis and monitoring of all conditions where M-protein is barely detectable and hardly quantifiable [10,18,19,20]. The introduction of sFLC measurement has since then emphasized the crucial role of an altered κ/λ ratio (sFLC ratio >1.65 or <0.26) as a predictor of progression from MGUS to MM [10,15,17]. 

Moreover, in the context of relapse, even a small amount of reactivated myeloma cells may produce a free light chain (FLC), whose levels may rise above detection limits before intact immunoglobulin is detected [21,22,23]. 

However, reverse phenomenon may occur during a favorable response to drug treatment. In such cases, FLC levels may decrease compared to previous immunoglobulin patterns. It is clear that sFLC quantification enables early detection of disease progression compared to SPE and immunofixation (IFE) [14,20].

Currently available methods for sFLC quantification are the Freelite™ assay (The Binding Site) and the N-Latex FLC (Siemens) [24,25,26]. Comparative studies conducted in order to evaluate the concordance between the two assays have produced varied results [20,26,27]. It is therefore recommended that the same method be used, especially for monitoring therapy responses [28,29,30,31,32,33,34].

In this study, we evaluated sFLC concentrations and their ratios in 85 samples of patients with MG, admitted to the National Cancer Institute “G.Pascale” (Table S1). The sFLC were measured using two immunological commercial kits in order to compare methods (Table S2). 

Differences in the results obtained from both methods were observed in three patients with plasma cell dyscrasias.

## 2. Case Reports

### 2.1. First Clinical Case

**Figure 1:**
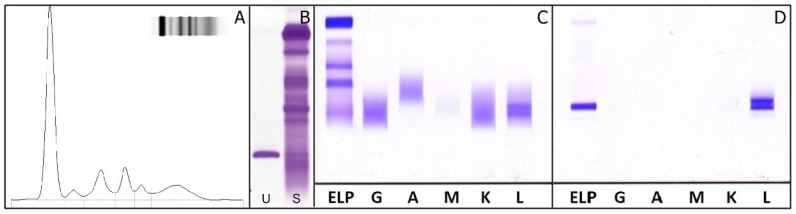
First clinical case: (**A**) Agarose gel electrophoresis of serum proteins, performed with semi-automatic analyzer Hydrasys 2 (Sebia), shows a normal profile; (**B**) High resolution electrophoresis performed on agarose gel in urine (U) and serum (S) samples of patient. In both matrices M-proteins were detected; (**C**,**D**) Immunofixation of serum (**C**) and urine (**D**). The figures show two monoclonal λ-free light chains proteins. ELP: fixative for electrophoretically separated proteins; G: IFE with antiserum anti-γ; A: with antiserum anti-α; M: with antiserum anti-μ; K: with antiserum anti-κ; L: with antiserum anti-λ.

A 47-year-old woman was referred to the Orthopedic Day Surgery at the National Cancer Institute “G.Pascale” of Naples, Italy, due to the presence of bone pain. Pelvis radio diagnostics revealed osteolytic lesions within the right hemipelvis. The patients showed mild anemia (Hb: 11.1 g/dL—Reference Range (RR): 12–16 g/dL) and hypercalcemia (11.2 mg/dL—RR: 8.6–10.2 mg/dL), while β2-microglobulin and creatinine were within RR. SPE was run on agarose gel (AG) (Hydragel 30 β1–β2, Sebia) using semiautomatic analyzer Hydrasys 2 (Sebia). No qualitative/quantitative alterations of serum proteins, particularly the γ-globulins, were detectable by SPE (Figure 1A). Nephelometric quantification of IgG, IgA, and IgM were performed on the BNP ProSpec nephelometer (Siemens Healthcare Diagnostics) according to the manufacturer’s instructions; all values were within RR. 

In accordance to the guidelines by the “International Myeloma Working Group” for the screening of plasma cell dyscrasias, sIFE (serum), uIFE (urine), and sFLC measurement were performed. 

**Figure 2:**
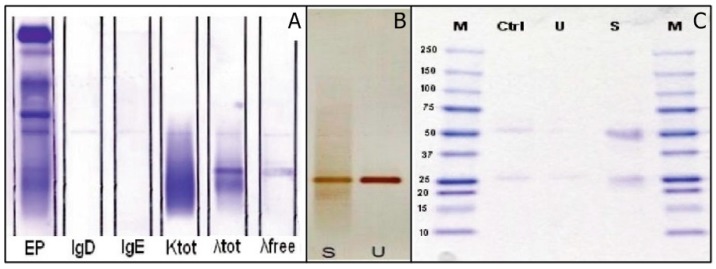
First clinical case: (**A**) Serum immunofixation of patients with anti-IgD, anti-IgE, and anti-λ free light chains antisera, which confirms the presence of an M-protein consisting of free light chains λ. EP: electrophoretic migration; IgD: IFE with antiserum δ; IgE: IFE with antiserum ε; Ktot: IFE with antiserum anti-κ; λ tot: IFE with anti-λ antiserum; λ free: IFE with specific antiserum anti-λ free; (**B**) Immunoblotting of serum (S) and urine (U) of the patient. The monoclonal protein is evident in both biological matrices; (**C**) SDS-PAGE is used for the separation of proteins based on their molecular weight. The bands are recovered after electrophoresis on agarose gel high-resolution. The figure shows the presence of proteins with MW of 22,000 and 44,000 Da, corresponding to monomers and dimers of λ free light chains. M: proteins PM-known index; Ctrl: control serum of patient with known monoclonal protein λ free light chain; U: urine of patient; S: serum of patient.

sFLC levels was quantified using two FLC assays: Freelite™ (The Binding Site) kits on the Cobas C 6000 immunoturbidimetric analyzer (Roche Diagnostics) and N-Latex FLC (Siemens Healthcare Diagnostics) using the BNP ProSpec nephelometer (Siemens). The Freelite™ was the first commercial method applicable on different nephelometers and turbidimeters, based on a mixture of polyclonal antibodies directed against different epitopes of FLC. Subsequently, the N-Latex FLC was available, but for exclusive use on nephelometers of the same manufacturer, based on a mixture of mouse monoclonal antibodies as probe [24,25,26]. Both tests were carried out as recommended by the manufacturers. sFLC quantifications using N-Latex FLC were within RR of the method, whereas sFLC λ concentration, detected using the Freelite™ kit yielded extremely high values with a very low κ/λ ratio (Table 1). Home-made high-resolution electrophoresis (HRE) performed on serum and urine samples, using a modified technique previously proposed and reported by Jeppsson et al. [35], showed the presence of an M-protein in the γ-region in both samples (Figure 1B). In order to identify suspicious M-proteins, previously detected by HRE, IFE (Hydragel IF 2/4, Sebia) was performed on both serum and 10× concentrated urine using the standard panel of antisera, which revealed the presence of two M-proteins, composed of λ light-chains in the absence of heavy chains (Figure 1C,D). The double bands were probably due to the same λ-chains present in the two states of aggregation: as monomers and as dimers. A subsequent sIFE was performed using anti-IgD, anti-IgE (Helena Laboratories), and anti-FLC λ (Dako) antisera, and analysis confirmed the presence of a λ FLC M-protein (Figure 2A). The presence of an M-protein, both in serum and in urine, was further confirmed by immunoblotting on nitrocellulose and detection with chromogen (“polyclonal rabbit anti-human λ free light chains” and “polyclonal goat anti-rabbit immunoglobulins” HRP, Dako; 3-ammino 9-etilcarbazolo, Helena) (Figure 2B).

The identification of the molecular weight (MW) of the M-proteins by SDS-PAGE (sodium dodecyl sulfate-polyacrylamide gel electrophoresis) confirmed that the M-proteins were composed of proteins weighing either 22,000 or 44,000 Da. There are two types of FLC: κ FLC, which may be found as circulating monomers weighing approximately 22.5 kDa, and circulating λ FLC, in the form of dimers, with a molecular weight of approximately 45 kDa. These results are concordant with the monomeric or dimeric conformation of the λ chains (Figure 2C). The bone marrow biopsy showed diffuse infiltration (>90%) of medium-sized plasmacytoid cells, with a positive immunophenotypic profile for CD138 and λ-markers; negative for CD20, cyclin D1, and panCK markers. Clinical and laboratory data were coherent with diagnosis of lambda light chain myeloma. The patient was hospitalized in the Hematology Oncology Unit. Within one month after therapy, both serum and urine IFE were performed, showing that M-protein disappearance and λ FLC quantification were within reference range.

### 2.2. Second Clinical Case

**Figure 3:**
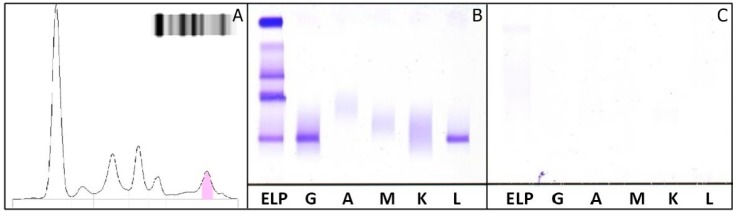
Second clinical case: (**A**) Agarose gel electrophoresis of serum proteins shows an M-protein migrant in the γ region; (**B**,**C**) Immunofixation of serum (**B**) and urine (**C**). IgG λ M-protein is detected in serum. ELP: fixative for electrophoretically separated proteins; G: IFE with antiserum anti-γ; A: with antiserum anti-α; M: with antiserum anti-μ; K: with antiserum anti-κ; L: with antiserum anti-λ.

A 34-year-old woman, with a diagnosis of MM, was referred to the out-patient department at the National Cancer Institute “G.Pascale” of Naples, Italy, for an annual follow-up. Laboratory results including hemoglobin, calcium, creatinine, and nephelometric quantification of IgG, IgA, and IgM were within RR, while the value of β2-microglobulin was high (2.80 mg/L—RR: 1.1–2.53). SPE showed an M-protein in the γ-globulin region (Figure 3A). 

In accordance with the guidelines of the International Myeloma Working Group, sIFE, uIFE, and sFLC were required. 

sFLC quantifications using N-Latex FLC were within RR, whereas κ FLC values, using the Freelite™ kit, yielded extremely low values with a low κ/λ ratio (Table 1). sIFE shows the presence of an IgG λ M-protein, whereas the uIFE (using 10× concentrated urine samples) shows the absence of M-protein (Figure 3B,C). 

### 2.3. Third Clinical Case

**Figure 4:**
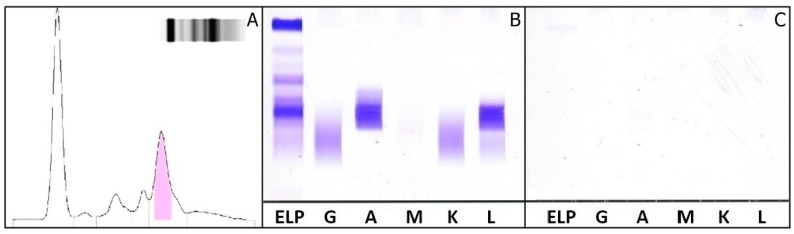
Third clinical case: (**A**) Agarose gel electrophoresis of serum proteins shows an M-protein migrant in β2 region; (**B**,**C**) Immunofixation of serum (**B**) and urine (**C**). IgA λ M-protein is detected in serum. ELP: fixative for electrophoretically separated proteins; G: IFE with antiserum anti-γ; A: with antiserum anti-α; M: with antiserum anti-μ; K: with antiserum anti-κ; L: with antiserum anti-λ.

A 59 year-old male was referred to the out-patient department of the National Cancer Institute “G.Pascale” of Naples, Italy. Various laboratory tests have been required by his primary care physician for a routine checkup. SPE revealed the presence of an M-protein in the β2-region (Figure 4A). Nephelometric quantification of IgG and IgM were within RR values, but increased IgA (25.70 g/L—RR: 0.70–4) was observed. sFLC quantifications by the N-Latex FLC assay showed values within RR, whereas the Freelite™ assay detected low κ FLC values, with a very low κ/λ ratio and elevated λ FLC values (Table 1). sIFE showed the presence of an IgA λ M-protein and an absence of M-protein in uIFE (Figure 4B,C). The bone marrow biopsy showed diffuse infiltration of medium-sized plasmacytoid cells, with a positive immunophenotypic profile for CD138 and λ-markers; negative for the cyclin D1 marker. 

The patient was hospitalized in Hematology Oncology Unit with diagnosis of MM.

## 3. Discussion

Eighty-five samples of patients with plasma cell dyscrasia were included in this study (Tables S1 and S2). Serum samples from each patient were analyzed for sFLC and calculated κ/λ ratio using two FLC assays (Freelite™ and N-Latex FLC) in order to compare methods. We have reported three particular cases with interesting differences in the results obtained from these two assays.

IMWG incorporated the measurement of sFLC into the guidelines for the detection of multiple myeloma and related disorders and defined the response criteria during treatment [14,17]. SPE, sIFE, and uIFE for the detection of urinary Bence Jones protein (PBJ) as well as the quantification of sFLC and their ratio are all necessary tools for patient management.

Among the three cases reported here, sFLC κ and λ were differently quantified by the two assays. In particular, in the first case, we observed no qualitative alterations of the serum protein pattern, and in the absence of the Freelite™ assay, sFLC measurement could not have highlighted the increase of λ FLC. 

In the second case, neither assay detected elevated λ FLC levels, so the abnormal κ/λ ratio is not due to an increase of λ FLC, but rather a result of suppression of κ FLC. 

In the third case, although SPE showed the presence of an M-protein, sFLC quantification with N-Latex FLC was within RR, not allowing for a correct sFLC measurement. In this study, the discrepancies in λ FLC measurement were probably related to the specificities and affinities of the antibodies used by the two methods. 

In patients affected by plasma cell dyscrasias, the sFLC quantification enables early monitoring of disease progression compared to both SPE and IFE [14,17]. The use of quantitative serum assays for κ and λ FLC has increased the sensitivity of serum testing strategies for identifying MG, especially the monoclonal light chain diseases [16]. 

The difficulties associated with the development of specific methods for measuring sFLC are mainly due to the uniqueness of monoclonal immunoglobulin and their modest serum concentration. Serum concentrations of circulating FLC may rise as a result of reduced clearance in the presence of renal failure, as well as upon increased FLC synthesis due to an overproduction of polyclonal immunoglobulins (as in the case of infectious and autoimmune diseases) or in the case of monoclonal B lymphocyte or plasma cell proliferation. The κ/λ ratio is generally used as a marker of monoclonality in the absence of concomitant diseases that affect kidney function.

A recent study investigates the possibility of using mass spectrometry (MS) to measure FLC and intact immunogloblulins. The preliminary results are very interesting since the method does not appear to suffer from the well-known analytical issues affecting the immunological methods for FLC detection, like antigen excess and non-linearity [36].

Our study demonstrated that there is only a moderate concordance between the two FLC assays (Tables S3 and S4). This implies that two FLC assays cannot be used interchangeably for the follow-up of patients affected by MG. Given the extreme structural heterogeneity of FLC in myeloma, it is possible that individual FLC may exhibit antigenic determinants which may, in turn, not be properly recognized by one of the antibody mixtures employed [20,26].

The three cases presented herein prove the non-interchangeability of the two commercial kits for sFLC measurement, confirming that patients should be monitored with the same assay. The Binding Site assay has been available for a longer time, and several studies suggest that Freelite™ has higher sensitivity than N-Latex FLC [20,33,37]. 

Our study raises a few clinically relevant points. Firstly, at diagnosis, the two FLC assays showed a high level of clinical concordance despite differences in FLC quantitation, whereas in two cases (Cases 1 and 3) the two FLC assays were clinically discrepant. Overall, from the patient screening, there was 96% agreement in patient diagnosis. Secondly, despite the high sensitivity of sFLC analyses and its proven utility, Case 2 demonstrates that occasionally certain patients can still be missed by this technique.

In conclusion, the current criteria for the diagnosis of myeloma suggest that SPE be used to detect M-protein and IFE used to characterize it. Indeed, the combination of SPE, IFE, and FLC provides relatively simple and sensitive diagnostic testing, which is needed in the management of these patients. 

## Figures and Tables

**Table 1:** The measurement of sFLC using N-Latex FLC and Freelite™ on serum in the three clinical case.

Cases	FLC	N-Latex FLC	Reference Range	Freelite™	Reference Range
First case	s-FLC κ (mg/L)	16.4	6.7–22.4	9.1	3.3–19.4
	s-FLC λ (mg/L)	23.0	8.3–27.0	**818.8**	5.7–26.3
	Ratio κ/λ	0.71	0.31–1.56	**0.01**	0.26–1.65
Second case	s-FLC κ (mg/L)	8.2	6.7–22.4	**1.92**	3.3–19.4
	s-FLC λ (mg/L)	15.2	8.3–27.0	18.27	5.7–26.3
	Ratio κ/λ	0.54	0.31–1.56	**0.1**	0.26–1.65
Third case	s-FLC κ (mg/L)	6.7	6.7–22.4	**2.06**	3.3–19.4
	s-FLC λ (mg/L)	19.0	8.3–27.0	**69.7**	5.7–26.3
	Ratio κ/λ	0.35	0.31–1.56	**0.03**	0.26–1.65

## Supplementary Materials

Supplementary materials can be found at www.mdpi.com/1422-0067/18/4/804/s1.
