# Supplementary material for: Analytical Criticalities Associated to Different Immunological Methods for Serum Free Light Chain Detection in Plasma Cell Dyscrasias: A Description of Particular Clinical Cases
Source: Int J Mol Sci. 2017 Apr 12;18(4):804. doi: 10.3390/ijms18040804 (PMC5412388; doi:10.3390/ijms18040804)
Supplement: Supplementary file 1 [file ijms-18-00804-s001.pdf]

Supplementary Materials: Analytical Criticalities Associated to Different Immunological Methods for Serum Free Light Chain Detection in Plasma Cell Dyscrasias: A Description of Particular Clinical Cases

Rocco Sabatino, Antonio Perrone, Marco Cuomo, Sandra Liotti, Vittoria Barchiesi, Monica Cantile and Ernesta Cavalcanti

Table S1. Clinical characteristic of patients included in the study.

Parameter	Number of patients
**Gender**	
Female	34/85
Male	51/85
**Age**	
Median (years)	67
>66	45
**Type of myeloma**	
MM	58/85
MGUS	27/85

MM: multiple myeloma; MGUS: monoclonal gammopathy of undetermined significance.

Table S2. Results of FLC κ/λ measurement and the calculated κ/λ ratio using Freelite™ and N-Latex assays.

Number	Sex	Age	N-Latex FLC κ mg/L (6.7–22.4)	N-Latex FLC κ mg/L (8.3–27)	N-Latex κ/λ FLC (0.31–1.56)	Freelite FLC κ mg/L (3.30–19.4)	Freelite FLC λ mg/L (5.71–26.3)	Freelite κ/λ FLC (0.26–1.65)
1	M	77	20.2	91	0.22	17.3	248.64	0.07
2	F	96	15	110	0.14	10.14	561.41	0.02
3	F	78	423	48.4	8.74	465.62	4.51	103.24
4	M	85	92.8	48.4	1.92	50.02	26.13	1.91
5	M	86	344	74.6	4.61	158.18	60.31	2.62
6	M	78	1100	7.99	137.67	1434.2	1.08	1327.96
7	M	73	15.5	74.7	0.21	9.82	41.43	0.24
8	F	67	7.58	1200	0.01	5.35	774.69	0.01
9	M	61	76.3	22.2	3.44	41.39	10.71	3.86
10	F	66	1530	2.98	513.42	2797	1.2	2330.83
11	F	68	51.3	13.6	3.77	41.31	8.87	4.66
12	M	67	58	9.05	6.41	31.49	1.63	19.32
13	M	56	10.7	969	0.01	7.88	80.63	0.10
14	M	57	9.12	1110	0.01	6.86	66.33	0.10
15	F	55	193	8.38	23.03	681.6	0.87	783.45
16	F	75	329	6.2	53.06	588	1.32	445.45
17	F	62	50.6	11.4	4.44	21.21	2.03	10.45
18	M	61	61.7	27.7	2.23	115.5	19	6.08
19	F	72	240	8.2	29.27	612.5	0.98	625.00

20	M	72	34.8	35.1	0.99	27.75	23.24	1.19
21	M	71	399	17.7	22.54	536.9	2.5	214.76
22	M	81	31.5	51.4	0.61	23.54	49.1	0.48
23	M	55	43.5	7.38	5.89	15.96	0.96	16.63
24	F	53	292	8.31	35.14	610	1.25	488.00
25	M	65	11.9	15.6	0.76	7.8	16.99	0.46
26	F	54	1550	6.34	244.48	1997.2	0.85	2349.65
27	F	63	48.4	17.6	2.75	40.05	8.04	4.98
28	F	73	10.1	58.5	0.17	8.57	552	0.02
29	M	69	22	69.1	0.32	15.9	30.78	0.52
30	F	70	967	22	43.95	1972	9.71	203.09
31	F	55	102	11.7	8.72	155.66	1.48	105.18
32	F	75	51.5	59.7	0.86	34.56	31.6	1.09
33	F	66	316	8.04	39.30	403.88	0.86	469.63
34	M	59	18	57.4	0.31	14.75	45.64	0.32
35	M	56	217	18.1	11.99	455.3	1.65	275.94
36	M	65	8.41	213	0.04	6.81	144.26	0.05
37	M	61	34.4	10.6	3.25	27.31	0.93	29.37
38	M	65	14.6	14.8	0.99	10.99	11.85	0.93
39	M	57	16.8	158	0.11	10.26	151.84	0.07
40	M	69	28.3	121	0.23	20.52	297.76	0.07
41	F	83	23.6	249	0.09	19.3	208.32	0.09
42	M	51	18.2	48.9	0.37	12.86	28.4	0.45
43	M	57	11.1	300	0.04	8.36	142.6	0.06
44	M	64	347	13.9	24.96	2044	7.34	278.47
45	F	34	8.2	15.2	0.54	1.92	18.27	0.1
46	M	63	30.2	12.1	2.50	30.08	9.38	3.21
47	M	68	20.7	34.2	0.61	14.78	18.26	0.81
48	M	57	22.6	2780	0.01	13.27	1460.6	0.01
49	M	80	115	24	4.79	159.75	12.66	12.62
50	M	77	47.8	60.1	0.80	55.09	59	0.93
51	M	77	9.48	1190	0.01	4.64	2673	0.001
52	F	36	13.1	7.57	1.73	10.8	1.59	6.79
53	M	78	52.3	51.9	1.01	73.53	20.89	3.52
54	M	67	32.6	6.22	5.24	219.75	7.61	28.88
55	M	67	15.3	135	0.11	11.93	139.34	0.09
56	M	66	29.5	18.3	1.61	19.94	10.81	1.84
57	M	84	11.8	36.4	0.32	8.32	26.9	0.31
58	F	69	11.4	233	0.05	9.05	85.72	0.11
59	F	46	16.4	23	0.71	9.14	818.8	0.01
60	F	55	11.2	262	0.04	7.14	1009.1	0.01
61	F	77	15.4	29.7	0.52	11.95	19.77	0.60
62	F	71	9.63	9.55	1.01	6.77	7.8	0.87
63	M	76	26.4	172	0.15	20.19	85.15	0.24
64	M	78	26.4	32	0.83	21.91	19.15	1.14
65	F	48	52.5	12.6	4.17	129.11	0.94	137.35
66	M	65	123	5.1	24.12	177.39	1.22	145.40
67	F	64	17.3	30.3	0.57	17.76	16.28	1.09
68	F	74	29.3	73.6	0.40	18.92	33.28	0.57
69	M	59	76.9	17.5	4.39	47.74	7.17	6.66
70	M	78	91.1	155	0.59	102.12	69.5	1.47

71	F	62	20.6	14.3	1.44	17.17	11.74	1.46
72	M	53	13.1	12.2	1.07	10.09	10.88	0.93
73	M	70	2690	18.9	142.33	4100	2.52	1626.98
74	M	78	11.4	19.6	0.58	10.16	11.18	0.91
75	F	75	28.3	31	0.91	19.41	22.13	0.88
76	F	74	225	23	9.78	439.66	9.6	45.80
77	M	64	60	24.5	2.45	38.24	11.37	3.36
78	M	59	6.7	19	0.35	2.06	69.7	0.03
79	M	57	6.51	12.5	0.52	4.99	9.05	0.55
80	M	66	34.8	40.5	0.86	26.05	17.67	1.47
81	M	67	31.6	21.1	1.50	36.79	19.01	1.94
82	F	67	16.5	16.3	1.01	9.03	9.49	0.95
83	F	65	271	15.5	17.48	52.35	1.09	48.03
84	F	71	68.3	8.92	7.66	102.38	1.51	67.80
85	M	76	22.6	28.5	0.79	14.81	18.88	0.78

Table S3. Median of FLC κ/λ measurement and the calculated κ/λ ratio using Freelite™ and N-Latex assays.

Patient	N-Latex FLC κ mg/L (6.7–22.4)	N-Latex FLC λ mg/L (8.3–27)	N-Latex κ/λ FLC (0.31–1.56)	Freelite FLC κ mg/L (3.30–19.4)	Freelite FLC λ mg/L (5.71–26.3)	Freelite κ/λ FLC (0.26–1.65)
MM + MGUS	30.2	23	1.01	21.21	16.28	1.19
MM	41.6	23	1.47	29.62	15.165	1.925
MGUS	18	24.5	0.86	14.78	16.28	0.93

Table S4. Comparison method between Freelite™ and N-Latex assays.

Patient	Number	Range N-Latex FLC mg/L	Range Freelite FLC mg/L	Slope Passing-Bablock. 95% CI	Intercept Passing-Bablock. 95% CI
MM + MGUS	85				
FLC K	85	6.51–2690	1.92–4100	0.77 (0.70–0.90)	6.11 (4.61–7.88)
FLC λ	85	2.98–2780	0.85–2673	1.34 (1.12–1.55)	6.41 (4.38–7.22)
κ/λ Ratio	85	0.01–513.42	0.001–2349	0.23 (0.17–0.35)	0.23 (0.14–0.34)
MM	58				
FLC K	58	6.51–2690	1.92–4100	0.75 (0.65–0.83)	7.52 (5.76–9.95)
FLC λ	58	2.98–2780	0.85–2673	1.18 (0.98–1.54)	7.08 (5.15–9.31)
κ/λ Ratio	58	0.01–513.42	0.001–2349	0.20 (0.11–0.26)	0.24 (0.14–0.33)
MGUS	27				
FLC K	27	8.41–292	6.7–610	1.15 (0.81–1.55)	1.44 (−2.37–5.58)
FLC λ	27	6.2–1110	0.94–552	1.60 (1.26–2.16)	1.12 (−8.17–5.56)
κ/λ Ratio	27	0.01–35.14	0.02–488	0.63 (0.25–0.80)	0.14 (−0.01–0.53)

CI 95% confidence interval.
